# Pathogen detection in central nervous system infections: moving metagenomic sequencing closer to clinical practice

**DOI:** 10.1186/s12879-026-13276-9

**Published:** 2026-06-11

**Authors:** Nicola Cumley, Josh Quick, Thomas Brier, Sam Wilkinson, Chris Kent, Zaki Hassan-Smith, Nicholas Loman, Ghaniah Hassan-Smith

**Affiliations:** 1 Institute of Microbiology and Infection, University of Birmingham, Edgbaston, Birmingham, Birmingham, B15 2TT UK; 2 Division of Medicine, University Hospitals Birmingham NHS Trust, Edgbaston, Birmingham, B15 2TH UK; 3 Department of Neurology, University Hospitals Birmingham NHS Trust, Edgbaston, Birmingham, B15 2TH UK; 4 Aston Medical School, Aston University, Birmingham, B4 7ET UK

**Keywords:** Metagenomics, Cerebrospinal fluid (CSF), Central nervous system infections (CNSI), Bioinformatics, Cell-free, Pathogen detection, Diagnostics

## Abstract

**Background:**

Central nervous system infections (CNSI) contribute significantly to global disability and mortality, but the causative agent is often undetected. Metagenomic sequencing offers the potential to enhance diagnostic sensitivity, particularly in cases of unusual or partially treated infections. However, caution is required in interpretation of metagenomics data due to technical artefacts from contamination or non-specific read mapping which can reveal a broad spectrum of biologically plausible but diagnostically unlikely organisms.

**Methods:**

This study compares the performance of metagenomic sequencing with standard clinical microbiology methods using cerebrospinal fluid (CSF) from patients with CNSI and non-infected control samples. To evaluate sensitivity of different laboratory approaches, we sequenced DNA and RNA metagenomic libraries extracted from CSF, using both cell-free and cellular fractions. We then devised a set of simple, easily interpreted yet rigorous filters tailored for clinical metagenomics to generate a framework for result interpretation that can be readily applied by clinical scientists.

**Results:**

We demonstrate that composite filtering strategies are essential to reduce misleading signals and support standardised workflows. Additionally, our results suggest that a cell-free sample preparation approach can improve confidence in identifying clinically relevant pathogens, highlighting the impact of sample preparation on results quality.

**Conclusion:**

In this study we describe a reproducible method that can be incorporated into a practical framework for clinical application of metagenomic sequencing in CNSI diagnostics.

**Supplementary information:**

The online version contains supplementary material available at 10.1186/s12879-026-13276-9.

## Background

Central nervous system infections (CNSI) include meningitides- caused by infection of the meningeal layers covering brain tissue and encephalitides- characterised by inflammation of brain tissue. The World Health Organization (WHO) has set a target to defeat meningitis by 2030, highlighting the global significance of CNSI [1]. Neurological complications resulting from inadequate or delayed treatment contribute to a disproportionately high burden of morbidity and mortality compared to systemic infections [2–5].

Standard testing for CNSI currently includes cerebrospinal fluid (CSF) Gram stain and culture, antigen detection tests, and pathogen-specific molecular studies, along with paired chemical and cellular analyses. However, these approaches can lack sensitivity or specificity as culture-based microbiology diagnosis may be obscured by prior initiation of antimicrobial therapy, ultimately affecting assurance of the final diagnosis [3, 5–8]. Detection rates generally fall below 50% for CNSI cases, with up to 70% of encephalitides remaining undiagnosed and positive pathogen identification in viral meningitis being as low as 4% [9–13]. Concurrently, cases of viral meningitis and encephalitis have increased, up to seven-fold between 2004 and 2013 in a UK-based study [14]. This trend can have adverse effects on antimicrobial resistance patterns due to widespread empiric treatment, as well as hospital-based outcomes, including length of stay [2]. Achieving precise and rapid diagnosis to inform targeted treatment remains an essential objective, reflected in recent UK guidelines that highlight use of molecular assays to enhance pathogen detection in the diagnosis and management of acute meningitis [15–18].

Metagenomic sequencing is appealing for infectious disease diagnostics due to its untargeted approach that can identify both known and unexpected pathogens. This has particular utility in neurological infections given established challenges in accurate pathogen identification within the CNS [8, 19–21]. Further, the whole-genome information obtained during sequencing can be utilised for evolutionary tracing, strain identification, and predicting drug resistance [22]. The use of metagenomic sequencing for diagnosing CNSI has seen increased popularity recently, with smaller case reports being followed by larger-scale validation and clinical studies to guide treatment [23–27].

However, diagnostic sensitivity of metagenomics is hindered by the high proportion of host-derived nucleic acid in the samples which may impact pathogen detection [26, 28–31]. To increase metagenomics sensitivity, host depletion or pathogen enrichment of samples for metagenomic sequencing can be achieved through a variety of approaches but commonly used host depletion techniques are less effective when using CSF due to its low biomass [25, 28–33]. Using cell-free DNA (cfDNA) for metagenomic sequencing simplifies sample processing and cell-free approaches have proven efficacy in enriching for pathogen nucleic acid in CSF [28, 34] and blood [35].

CSF is low biomass and therefore metagenomic sequencing results are also prone to contamination, which can lower confidence in the results [8]. To address this, bioinformatic thresholds are typically employed [29]. For example, the SURPI+ analysis pipeline for pathogen identification [26] considers the number of reads of a pathogen, its proportion, normalisation to negative controls, and genome coverage of an organism.

Despite the promising evidence around the use of metagenomics in CNSI, there is little agreement around both the best sample preparation approaches, and little justification for choices around bioinformatics pipeline selection and filtering thresholds. To address this, we decided to benchmark the use of cell-free DNA, cell-free RNA, DNA extraction from cell pellets and RNA extraction from cell pellet to identify their relative performance. In order to accurately characterise the background contaminating sequences and to inform a clinical bioinformatics workflow we employed both standardised control samples, as well as control samples from individuals who did not have CNSI. This study proposes a practical metagenomic framework that brings the field closer to a clinically actionable approach for the diagnosis of CNSI.

## Methods

### Patient recruitment

Inclusion criteria for both infectious and non-infectious cases were patients aged greater than 18 years old, presenting acutely to hospital with symptoms requiring lumbar puncture (LP) for cerebrospinal fluid (CSF) analysis. Patients with suspected CNS infection (CNSI) clinically were identified by the study physician investigators, following acute admission under internal medicine or neurology at Queen Elizabeth Hospital, Birmingham, UK using British Infection Association (BIA) guidelines [15]. In patients presenting with suspected CNSI, this was defined as a compatible clinical syndrome (fever, meningism, altered mental status, or focal neurological deficit) together with abnormal CSF parameters (white cell count > 5 cells/µL and/ or elevated protein) and/or microbiological confirmation from CSF or blood.

Patients with opening pressure where measured of 12–20 cm H2O, clear and colourless appearance, CSF protein count < 0.4 g/L, CSF glucose 2.6-4.5mmol and CSF white cell count (WCC) < 5 cells/µL, with non-infectious aetiology were identified as control cases and classified as other neurological diseases (OND). This group comprised patients undergoing LP for reasons other than suspected CNSI, primarily for headache and to exclude vascular e.g. subarachnoid haemorrhage or structural causes for clinical presentation. Supplementary information Table 2 details clinical diagnosis and cell counts where available for recruited patients.

Written, informed consent to obtain samples following standard clinical diagnostic protocols was obtained from patients or surrogates (for patients who lacked decision-making capacity) under Human Biomaterials Resource Centre (HBRC), University of Birmingham ethical approval (21–396), by study physician investigators.

CSF samples were obtained using routine protocols by the internal medicine and neurology teams and transported to the Biobank after collection. Samples were logged, anonymised and frozen at -80^°^C, for storage in secure CL2 research laboratories at the University of Birmingham. Allied clinical data was stored in secure, password-protected files under standard data quality and management processes in accordance with University Hospitals Birmingham policy, and the analytical development work was performed blinded, with clinical microbiological diagnoses not released to the laboratory research team until final analysis performed.

## Nucleic acid extraction

0.5-4 ml of frozen CSF was thawed and spun at 3000x*g* for 15 min. Total nucleic acid was extracted from the supernatant using the QIAamp ccfDNA/RNA kit according to the kit instructions. DNA/RNA was eluted in 50 µl of nuclease free water. The cell pellet was retained and frozen at -80^°^ C for later use. Cell pellets were defrosted and resuspended in 750 µl of DNA/RNA shield, added to a ZR BashingBead Lysis Tube and extracted using the ZymoBIOMICS DNA/RNA Miniprep Kit (Zymo Research). Cell pellet samples were lysed using the MPBio fastprep instrument with the following protocol: 6 m/s for 40s, repeated four times with samples resting for one minute on ice in between each agitation. Samples were eluted in 50 ml of nuclease free water.

The 50 µl elute from both extraction methods was split into ‘RNA’ and ‘DNA’ fractions and 25 µl stored at -80 °C for cDNA synthesis and SMART-9 N. Each extraction batch contained a positive control and a negative control. Nuclease free water was used as the negative control.

The positive control for the cell-free extractions comprised of fragmented DNA, from the ZymoBIOMICS microbial community standard (D6300) and fragmented encephalomyocarditis virus (ECMV) RNA spiked into an OND sample chosen at random. The positive control for the cell pellet extractions comprised of the ZymoBIOMICS microbial community control and ECMV RNA spiked into the cell pellet of the same OND sample used for the cell-free control. Each extraction batch also included at least one OND sample.

## SMART-9 N

Given the importance of RNA viruses in causing CNSI we used the SMART-9 N protocol [36] to enrich for RNA in both the cell-free and the cell pellet fractions. After defrosting the RNA fraction, 25 µl of total nucleic acid elute from both cell-free and cell pellet methods was DNAse treated using Turbo DNAse (Invitrogen), cleaned up using a Zymo clean and clear concentrator-5 and eluted in 15 µl of nuclease-free water.

10 µl of extracted total RNA from each sample was annealed with 1 µl of NEB-RT-9 N (2 mM, AAGCAGTGGTATCAACGCAGAGTACNNNNNNNNN) and 1 µl of dNTPs (10 mM) at 65 °C for 5 min. The annealed RNA was cooled to 4 °C on ice before adding the following: 4 µl of Superscript IV buffer, 1 µl 0.1 M DTT, 1 µl Rnase OUT, 1 µl of the RNA oligo NEB-SSP (2 mM, GCTAATCATTGCAAGCAGTGGTATCAACGCAGAGTACATrGrGrG) and 1 µl Superscript IV (Thermo Fisher). This was then incubated at 42 °C for 90 min followed by 70 °C for 10 min. 10 µl of cDNA was mixed with 25 ml of 2xQ5 mastermix (New England Biolabs), 2 µl of NEB-PCR (20 mM, AAGCAGTGGTATCAACGCAGAGT), and made up to 50 µl with water for amplification. Cycling conditions were as follows; 90 °C for 45s, 30 cycles of 98 °C for 10s, 65 °C for 15s, 72 °C for 3 min, then a final extension at 72 °C for 5 min and hold at 4°C^55^.

## Library prep

DNA and cDNA were quantified using the Qubit™ fluorescent dye (Invitrogen) on a BMG CLARIOstar (Excitation 483 − 14, Emission 530 − 30). DNA and cDNA from the cell-free fraction were prepared for sequencing using the NEBNext Ultra II kit following the kit protocol with a barcoding PCR of 10 cycles for DNA and 7 cycles for cDNA. Nucleic acid from the cell pellet fraction was fragmented and prepared for sequencing using the NEBNext Ultra II FS kit with barcoding cycles of 6 cycles for DNA and 5 cycles for cDNA. Libraries were quantified using a TapeStation (Agilent).

DNA and cDNA libraries were separately pooled with equal concentrations of each barcode. The cell-free fragment libraries were sequenced on an Illumina HiSeq 2000 employing the paired-end 250 bp rapid run mode. The cell pellet fraction experiment was run on an Illumina NextSeq 2000 employing the paired-end 150 bp run mode using a P3 cartridge.

## Bioinformatics

FASTQ files taken from Illumina sequencing were first quality trimmed using Trimmomatic (SLIDINGWINDOW:4:20 MINLEN:30 ILLUMINACLIP: only-TruSeq3-PE-2.fa:2:40:15) [37]. Primer sequences were removed from cDNA sequences using Cutadapt [38]. FASTQ files were initially host-depleted by aligning to the human reference genome GRCh38 using Minimap2 and discarding mapping reads to generate dehosted results [39]. Resulting BAM files were converted to FASTQ using samtools [40]. KrakenUniq [41] was used to provide taxonomic classification, a unique *k*-mer count and coverage for reads mapping to species- level assignments in each sample. The kraken_extract.py script from the KrakenTools suite (https://github.com/jenniferlu717/KrakenTools*)* was used to extract reads mapping to individual taxonomic assignments [42], and the closest mapping species were identified through BLAST [43]. Confirmatory alignments for every taxonomic assignment deemed to be significant by filtering methods was performed using minimap2 [39] by aligning dehosted FASTQ files against a reference pathogen genome. BAM files were visualised using Tablet [44]. The rolling mean coverage depth for each genome position was calculated using a sliding window of 5000 bp for Neisseria and 150 bp for Human herpesvirus 7 (HHV-7). Coverage across the genome was visualised using ggplot2 [45].

### Analysis

Species level read assignments were normalised to reads per million (RPM).

$$\:RPM=\frac{Number\:of\:organism\:specific\:reads\:in\:the\:sample}{(Total\:number\:of\:reads\:in\:the\:sample/1{0}^{6})}$$

RPM was used to calculate relative RPM (rRPM) for each organism hit [19].

$$\:rRPM=\frac{RPM\:of\:organism\:in\:the\:sample}{RPM\:of\:organism\:in\:the\:negative\:control}\:$$

The minimum RPM in the negative control was set to 1. The negative control used was specific to the extraction batch for each sample. For additional confidence that an organism is likely to be in a sample we calculated an *E*-value (here referred to as *E-*score in order to avoid confusion with BLAST’s *E-*value) [46]:

$$\:E-score=\left(\frac{Kmers}{reads}\right)x\:coverage$$

Where coverage is:

$$\:Coverage=\frac{kmers}{genome\:size}$$

For a bacterial and fungal species to be considered a significant taxonomic assignment the *E*-score had to be > 0.001, RPM > 10 with at least three non-overlapping regions of the genome present when reads were mapped to a reference genome. For viral identification and *Mycobacterium tuberculosis*, a single KrakenUniq classified read with no reads of the same taxonomic assignment in the negative control in that batch was sufficient to be considered significant for mapping to the reference genome, where three discrete mapping regions had to be visualised [20, 47]. Reference genomes used can be viewed in Supplementary Table 5.

For comparison of assignments to results reported on each patient from the clinical microbiology laboratory, the assigned pathogen additionally had to be present on an allowed taxon whitelist of 117 CNSI expected organisms (Supplementary Table 1) [15, 48–50].

An accompanying Python script, BigBugData (source code available at https://github.com/tombch/bigbugdata) was used to process the KrakenUniq reports, which gave a read- out of the top assignments in each sample with RPM, rRPM and *E-*score calculated. Additionally BigBugData filtered aggregated data from each sample against organisms present in the allowed taxon whitelist.

## Results

### Patients

32 patients were recruited between October 2017 and February 2019. Of these, 15 patients met the criteria of CNSI with a total of 25 samples being obtained. Two patients (P01 and P11) had two samples received for the study and patient 8 had nine samples received for the study. Where more than one sample was received from a patient this represented separate aliquot’s obtained from a single lumbar puncture procedure. All 25 CNSI samples, from 15 patients, were included in the final analysis.

The remaining 17 patients did not have evidence of CNSI or other neuroinflammatory disease and were categorised as ‘other neurological diseases’ (OND), a single CSF sample was received from each of these patients. One of the OND samples was used for early stage validation and so excluded from the study. Samples from four OND patients were used as a background matrix for spike- in positive controls. After sequencing it was observed that one OND sample was contaminated in the cell free extraction and so all fractions from that patient were excluded from analysis. This left samples from 11 OND patients included in the final sequencing analysis.

Each extraction batch contained a positive control, and at least one negative control.

**Fig. 1:**
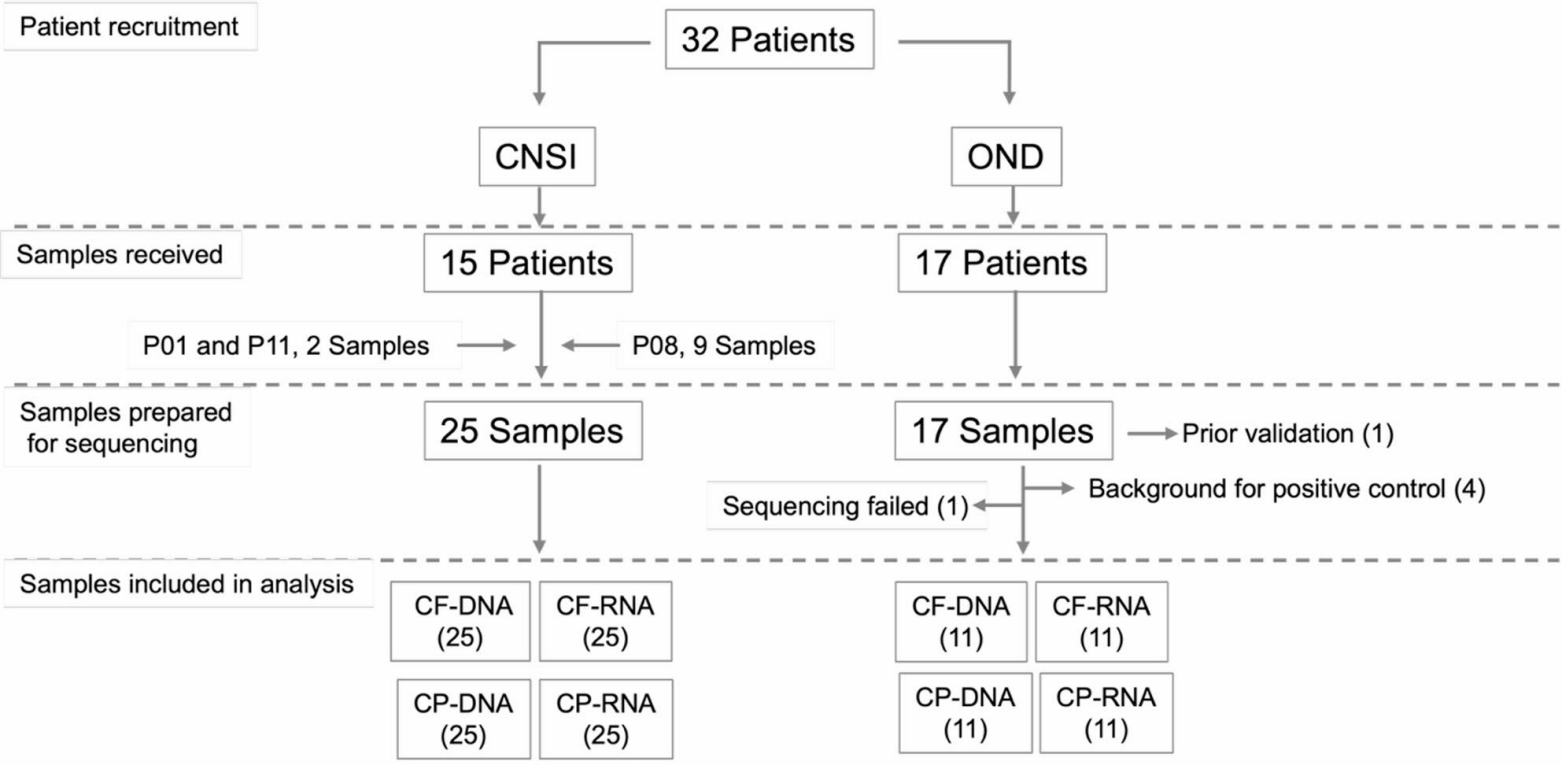
Patient recruitment and samples received. Patients recruited into the study were coded CNSI or OND based on clinical symptoms and initial pathology. Three patients coded CNSI had multiple samples received from the same lumber puncture. Six of the samples received from the 17 OND patients were excluded from the final analysis. DNA and RNA libraries were prepared from the cell free and cell pellet fractions of 25 samples from 15 recruited CNSI patients and 11 samples from the 17 recruited OND patients

### Sequencing results

cDNA and DNA libraries were prepared for both the cell-free and cell-pellet fraction for each of the 36 samples and ten controls (i.e. 184 libraries in total). All samples had a library sequenced. Pooled sample libraries were purified and sequenced. Cell-free fraction libraries were sequenced on the Illumina HiSeq2000. Cell-pellet fraction libraries were sequenced on the Illumina NextSeq 2000. One OND sample was excluded from final analysis due to contamination in the cell free fraction observed after sequencing. The data for this study have been deposited in the European Nucleotide Archive (ENA) at EMBL-EBI under accession number PRJEB81291 following host de-contamination (https://www.ebi.ac.uk/ena/browser/view/PRJEB81291).

Determining thresholds for filteringKrakenUniq produces a large number of false positive taxonomic assignments which require filtering to make them interpretable [51]. Analysis of the cell-free sequencing of a mock community spiked into an OND sample was used to validate a filtering strategy for KrakenUniq assignments.

**Fig. 2:**
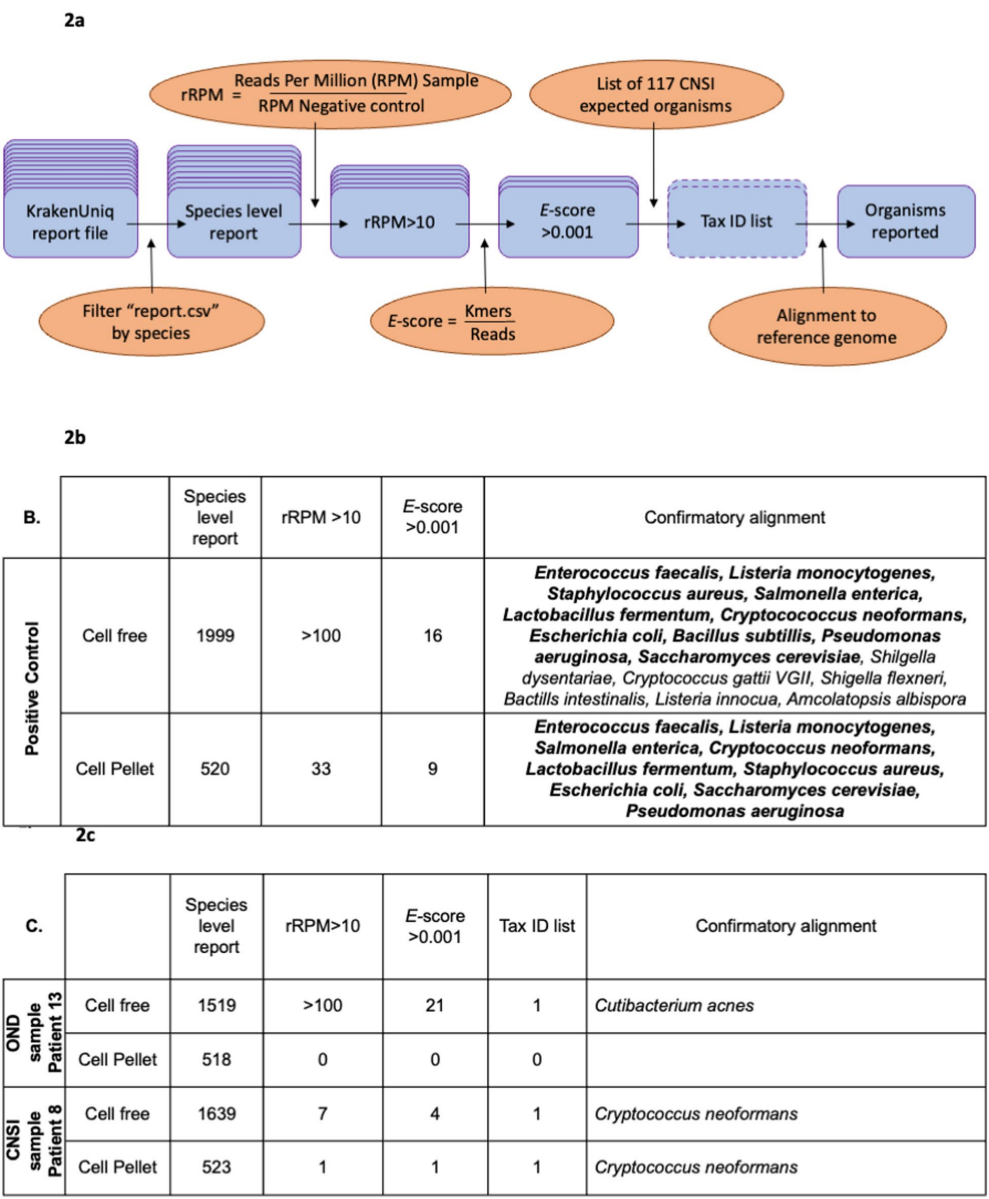
Sequential thresholding increases reliability of pathogen detection. For an organism to be considered detected in a sample using metagenomics, reads from the sample had to align to a reference genome in three discrete regions. To facilitate this, initially a series of bioinformatic thresholds were applied to each krakenUniq report to select assignments that were considered significant in the sample. The sequential steps are illustrated in schematic A. The kraken Uniq report was filtered to only included species level assignments, rRPM was calculated with bacterial and fungal assignments with a rRPM of over 10 considered as passing threshold. The E-score provided an additional level of stringency taking into account the coverage breadth and Uniq kmers. For direct comparison to clinical microbiology reports assignments also had to be present on the allowed taxon whiltelist, comprised of 117 organisms expected to cause CNSI. Tables B and C show the reduction in assignments at each step of the threshold process when using the positive control (**B**) and two patient samples (**C**). The number of assignments at each step is detailed in the columns of tables B and C, the final column lists the names of organisms which passed all the filtering steps and when aligned to a reference genome showed > 3 discrete regions of homology

Initial filtering used a previously described threshold based on > 10 rRPM [26]. However, despite using a rRPM threshold > 10 there remained > 100 assignments from the mock community of 12 organisms (Fig. 2b). Combining rRPM with an *E*-score > 0.001 reduced the number of assignments in the cell-free fraction to 16, (Fig. 2b). An *E-*score of threshold 0.01 has previous described by *Guellil et al.* [46]. All ten organisms expected in the ZymoBIOMICS microbial community standard were detected when the combined filters were used along with six additional unexpected assignments. Of these, five were closely related to the organisms expected (e.g. *Escherichia coli* vs. *Shigella dysenteriae*/*flexneri*), indicating likely misassignments by KrakenUniq. The sixth assignment was likely a contaminant, *Amycolatopsis albispora* is an organism found in the environment [52].

The same process was applied to a cell pellet fraction spiked with the same mock community, 33 assignments were present after rRPM filtering which fell to nine after applying the *E*-score threshold. The expected assignment to *B. subtilis* was present in the initial assignments but fell below the *E*-score threshold of 0.001, due to low genome recovery of the organism (Fig. 2b).

The utility of the *E*-score increased in samples where the proportion of reads that were not clinically significant comprised a higher proportion of the total number of reads. Figure 2c shows the effect of applying the combined filters on patient samples. Patient 13 (an OND case) had > 100 hits detected in the cell free fraction after the rRPM threshold had been applied. This reduced to 22 when the *E*-score was used. However, the *E*-score was of limited additional value in samples where a single pathogenic organism was present When these filter’s were applied to the sequencing output of both fractions of Patient 8, a patient with a microbiological diagnosis of *Cryptococcus neoformans*, the rRPM alone was effective at removing false positive assignments with the E-score only being required in the cell-free fraction (Fig. 2c).

Appling combined filters and thresholds to a mock community and true samples provides confidence that an E-score threshold of 0.001 and rRPM > 10 gave the optimal detection, reducing false positive assignments whilst allowing the identification of organisms known to be present in the sample. These steps were incorporated into a filtering process used for all clinical samples (Fig. 1a). Unfiltered assignments on every sample are available in Supplementary Table 4.

### Employing an allowed taxon whitelist of clinically expected organisms increased specificity

We also explored using an allowed taxon list of 117 expected CNSI organisms as an additional filtering approach, the list is available as supplementary Table 1. In the cell-free fraction of patient 8 (Fig. 2c), reads mapping to four species *Cryptococcus neoformans*, *Aspergillus glaucus*,* Prevotella melaninogenica*,* Psychrobacter sp.* P11G3 were present with a rRPM > 10 and *E-*score > 0.001. *Cryptococcus neoformans* is the most likely causative organism, with other hits arising from background contamination either from environment, patient or kits contaminants [53]. Including the allowed taxon whitelist increased the specificity of the assay for the purpose of clinical decision making.

### Pathogen- specific reads are enriched in the cell-free fraction

To estimate the enrichment potential of the cell free fraction, individual samples were considered. Coverage breadth, reads per million and total number of reads were compared between fractions in samples where the same assignment was seen passing filters in both fractions, irrespective of the taxon allowed whitelist or patient number.

**Fig. 3:**
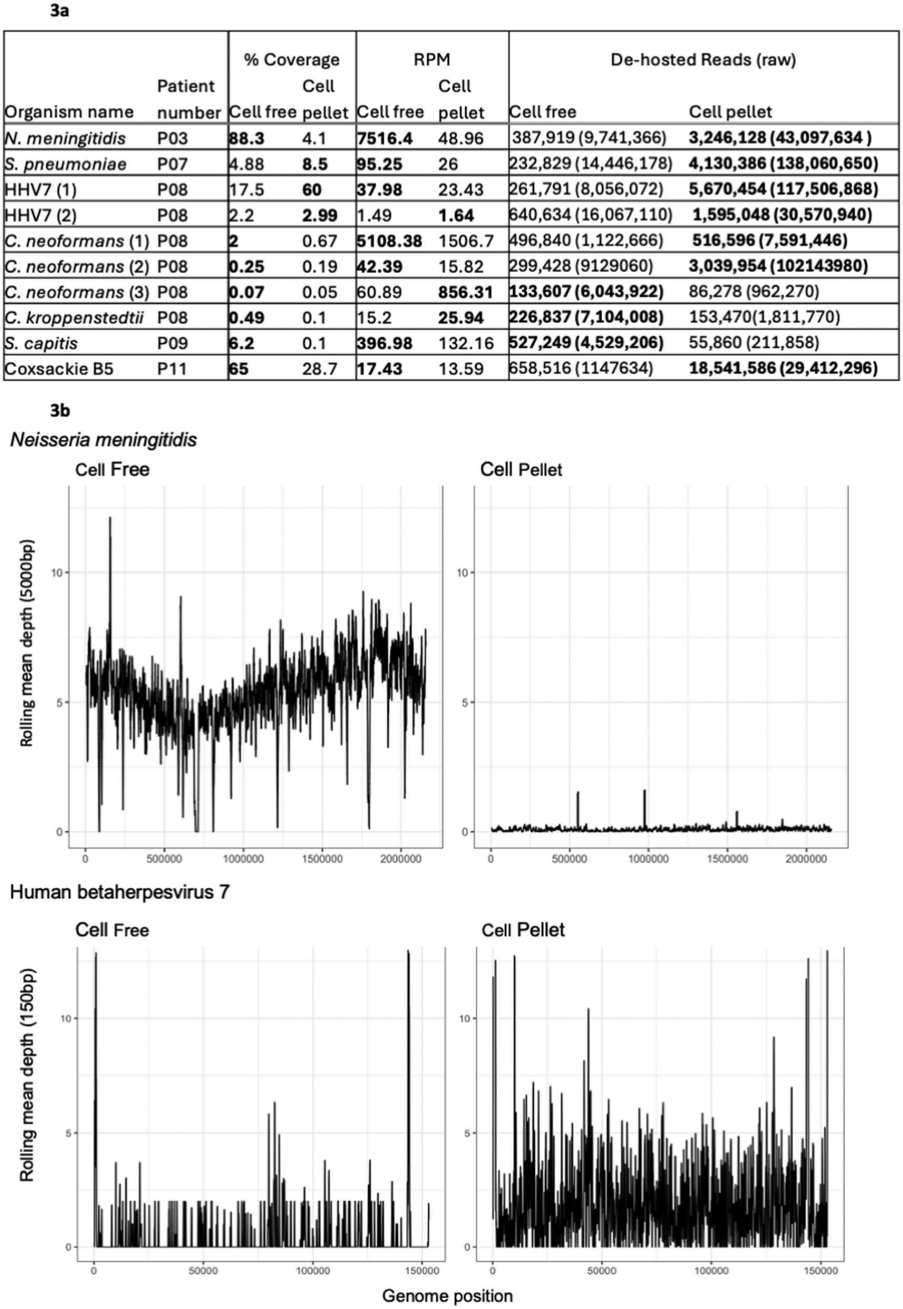
Horizontal genome coverage is greater in the cell-free vs. cell pellet fraction in patients with CNSI. When the same assignment was identified in both the cell-free and cell-pellet fraction of a sample the coverage breadth, normalized reads per million and de-hosted reads were compared for that organism between the two fractions, shown in the Table (**A**). Coverage plots (**B** and **C**) are shown for the organisms with the highest coverage in each fraction. Neisseria meningitidis (**B**) had the highest coverage in the cell-free fraction, HHV-7 (**C**) in the cell-pellet fraction

Enrichment of pathogen reads was determined using RPM and genome coverage. This comparison was possible in ten of 41 samples from five patients with only one sample from patient nine having multiple organisms in both fractions. Using RPM, eight of the 11 organisms found in both extraction methods were enriched in the cell-free fraction. Three organisms; *C. neoformans* (2), *C. kroppenstedtii* and HHV-7(1) were enriched in the cell-pellet fraction (Fig. 3). We did not observe any correlation with organism or patient for those assignments which were enriched in the cell-pellet fraction.

### Breadth of coverage is enriched in the cell-free fraction

We observed that for assignments passing filters in both the cell free and cell pellet fraction of an individual sample the breadth of coverage was often greater in the cell-free fraction. This is independent of RPM and the total number of de-hosted reads which were skewed in some cases by a high depth of coverage in localised regions. This is likely due to PCR duplication of a small number of input molecules. Considering RPM alone, Fig. 3a illustrates how *C. neoformans* (replica 3), *C. kroppenstedtii* and HHV-7 (replica 2) were enriched in the cell-pellet fraction, yet when considering coverage *C. neoformans* (replica 3) and *C. kroppenstedtii* were enriched in the cell-free fraction. Contrastingly, HHV-7 (replica 1) and *S. pneumoniae* had a higher coverage in the cell-pellet fraction but higher RPM in the cell-free fraction. The sample containing *S.pneumoniae* had the second highest cell count and the lowest proportion of microbial reads of all samples in the whole dataset, this may of compromised genome enrichment in the cell free fraction. Cell counts and read numbers can be viewed in supplementary Table 2. HHV-7 can persist within host cells in the absence of active infection [54]. Persistence of the virus within host cells could explain the higher genome recovery of HHV-7 from the cellular portion of the sample. The highest coverage depth for the cell-free fraction was *Neisseria meningitidis* (Fig. 3b) and for the cell-pellet fraction was HHV-7 (Fig. 3c). For *N. meningitidis* this represented 88% breadth and 5.4x depth and for HHV-7 this represented 60% breadth and 7.2x depth.

### Comparison to clinical microbiology results

**Table 1:** Comparison of organisms reported using clinical metagenomics with the clinical microbiology report in patients with signs of central nervous system infection (CNSI)

Patient	Clinical Microbiology	Cell free	Cell pellet
P01	Nothing reported ^[1]^	*Streptococcus sanuinis* ^*[2]*^	No assignments met reporting criteria
P03	*Neisseria meningitidis*	*Neisseria meningitidis*	*Neisseria meningitidis*
P04	Nothing **reported**	No assignments met reporting criteria	No assignments met reporting criteria
P05	Enterovirus	No assignments met reporting criteria	Enterovirus B ^[3]^
P07	*Streptococcus pneumoniae*	*Streptococcus pneumoniae*	*Streptococcus pneumoniae*
P08	*Cryptococcus neoformans*	*Cryptococcus neoformans*, HHV-7, HHV-4	*Cryptococcus neoformans*, HHV-7
P09	Enterovirus	*Staphylococcus aureus*,* Staphylococcus capitis*,* Staphylococcus haemolyticus*	*Staphylococcus capitis*,* Staphylococcus haemolyticus*
P10	Nothing reported	HHV-4	No assignments met reporting criteria
P11	Enterovirus	Enterovirus B	Enterovirus B
P12	*Staphylococcus warneri*	No assignments met reporting criteria	No assignments met reporting criteria
P15	Nothing reported	*Cutibacterium acnes*,* Staphylococcus epidermidis*	No assignments met reporting criteria
P29	Nothing reported	No assignments met reporting criteria	No assignments met reporting criteria
P30	Enterovirus	Enterovirus A	No assignments met reporting criteria
P31	Nothing reported	No assignments met reporting criteria	No assignments met reporting criteria
P32	Nothing reported	No assignments met reporting criteria	No assignments met reporting criteria

^[1]^ .Nothing reported - No organism were reported by the clinical microbiology laboratory. No assignments met reporting criterial – No species detected in the metagenomic sequencing met the criteria for reporting

^[2]^ Bacterial and Fungal organisms reported by metagenomic sequencing were present on an allowed taxon whitelist list, had an rRPM > 10, *E-score* of > 0.001 and at least 3 distinct areas of coverage when aligned to a reference genome. Viral organisms were reported if they were present on the allowed taxon whitelist list and had at least 3 reads in distinct parts of the genome when aligned to the reference genome and were not detected in the negative control

^[3]^ Nomenclature is consistent with the KrakenUniq report and the report available from the clinical microbiology laboratory

We compared the organism reported by clinical microbiology and the organism reported by clinical metagenomics (Table 1). To simplify the analysis individual samples were not considered, comparison is performed on a patient basis. Eight of the 15 (53%) patients coded CNSI had a positive microbiology result reported, in line with the local and national positivity rate [10]. Organisms found included *N. meningitidis*, Enterovirus, *S. pneumonia*,* C. neoformans* and *Staphylococcus warneri.* With the exception of *S.warneri* all these organisms are common causes of CNSI. Where multiple samples were processed for metagenomic sequencing on a single patient all assignments from every sample were considered, if one sample had the same species reported as the clinical microbiology report this was deemed concordant. Metagenomic sequencing was in agreement with clinical microbiology in five of the eight patients (62%) (Table 1). Four out of eight patients (50%) had the same organism detected in both cell-free and cell-pellet. One patient (P05) had an enterovirus detected only in the cell-pellet fraction and another patient (P30) had an enterovirus detected only in the cell-free fraction. Read counts, rRPM and E-score of each assignment are available in supplementary Table 3.

### Discrepancies between metagenomics and clinical microbiology

Metagenomics failed to detect enterovirus reported by the microbiology laboratory in one patient (P09) using both methods. Enterovirus was also not detected in one patient (P05) in the cell-free fraction and one patient (P30) in the cell-pellet fraction. Enterovirus was not found even in the unfiltered assignments. *Staphylococcus warnerii* identified by enrichment culture was not detected in either fraction and also not found in the unfiltered assignments for either fraction. HHV-7 was detected in both the cell-free and cell-pellet fractions and HHV-4 was detected in the cell-free fraction for one patient (P8) neither of which were reported by clinical microbiology. Mixed *Staphylococci spp.* were detected by metagenomics in both the cell-pellet and cell-free fractions for one patient (P9). Clinical microbiology instead reported e.

Enterovirus, but neither were detected by the other method. The cause of this is unknown and would need a clinical review to establish the significance of the findings.

### Increased diagnostic potential of cell-free sequencing

Three patients (P01, P10, P15) had no positive microbiology reported yet did have an organism detected with metagenomics in the cell-free fraction. Additional organisms that were detected by metagenomics included *Streptococcus sanguinis* (P01), HHV-4 (P10), *Cutibacterium acnes* and *Staphylococcus epidermidis* (P15) none of which were reported by clinical microbiology and could be considered normal flora as well as potentially pathogens. OND samples were not expected to harbour infectious organisms but pathogens were detected by metagenomics in two out of eleven patients (18.2%) (Table 2). The organisms detected were *C. acnes* (P13) and *S. epidermidis* (P14). All additional organisms detected by metagenomic sequencing were detected in the cell-free fraction rather than the cell-pellet fraction.

**Table 2:** Comparison of organisms reported using clinical metagenomics with the clinical microbiology report in patients with other neurological disease (OND)

Patient	Clinical Microbiology	Cell free	Cell pellet
P02	Nothing reported ^[1]^	No assignments met reporting criteria	No assignments met reporting criteria
P06	Nothing reported	No assignments met reporting criteria	No assignments met reporting criteria
P13	Nothing reported	*Cutibacterium acnes* ^[2]^	No assignments met reporting criteria
P14	Nothing reported	*Staphylococcus epidermidis*	No assignments met reporting criteria
P16	Nothing reported	No assignments met reporting criteria	No assignments met reporting criteria
P17	Nothing reported	No assignments met reporting criteria	No assignments met reporting criteria
P18	Nothing reported	No assignments met reporting criteria	No assignments met reporting criteria
P19	Nothing reported	No assignments met reporting criteria	No assignments met reporting criteria
P21	Nothing reported	No assignments met reporting criteria	No assignments met reporting criteria
P23	Nothing reported	No assignments met reporting criteria	No assignments met reporting criteria
P25	Nothing reported	No assignments met reporting criteria	No assignments met reporting criteria

^[1]^ Nothing reported; no organisms were listed in the clinical microbiology report. No assignments met reporting criteria; no species detected in the metagenomic sequencing met the criteria for reporting

^[2]^ Bacterial and Fungal organisms reported by metagenomic sequencing were present on anallowed taxon whitelist, had an rRPM > 10, *E-score* of > 0.001 and at least 3 distinct areas of coverage when aligned to a reference genome. Viral organisms were present on the allowed taxon white list had at least 3 reads in distinct parts of the genome when aligned to the reference genome and were not detected in the negative control

## Discussion

This study offers a novel, reproducible approach to metagenomic sequencing of clinical samples. We devised a practical yet robust bioinformatics filtering strategy to control false positives and maximise interpretive confidence for metagenomic sequencing data. Our method integrates established bioinformatic metrics— relative reads per million (rRPM) [19], unique *k*-mers [38], read counts and E-score thresholds [46] - to create a principled, easily interpretable and verifiable analysis framework. A key strength of this study was the inclusion and comparison of CSF samples from patients with diagnoses other than CNSI.

The untargeted nature of metagenomic sequencing is its major strength but also limits adoption for routine diagnostics due to the potential for detecting clinically irrelevant organisms such as low-abundance contaminants and normal human flora. In this work initial taxonomic classification resulted in many false positive assignments emphasising the need for enhanced filtering. Distinguishing true signals in metagenomics data from background noise is crucial, particularly when dealing with low-biomass samples like CSF where contaminating nucleic acids may disproportionately dominate the reads [55]. Using the metagenomics classifier KrakenUniq [41] which outputs a unique k-mer coverage metric for each assignment, we were able to improve reporting specificity by combining unique k-mer counts and read abundance. Simple thresholds were evaluated using a mock community to ensure that false positive hits were removed whilst still detecting expected organisms. The addition of a comprehensive allowed taxon list of 117 organisms added a further layer of specificity. Individual thresholds found to be effective in this study are the same as previously described [26, 46], we found that combining thresholds and the inclusion of an allowed taxon whitelist allowed for robust reporting of results. Using the reporting thresholds we only detected two assignments in samples from patients with OND. Clinical review of the OND cohort confirmed that none of the patients with detected pathogens had intracranial devices or a history of neurosurgical intervention, nor was there any clinical suspicion of healthcare-associated central nervous system infection. This supports interpretation of these findings as skin contaminants which could have been acquired at any point in the process from sampling to sequencing, rather than true pathogens. The few organisms reported in OND samples demonstrates the power of the filtering approach that was used.

Our method is simple compared to other methods such as Metamix, which employs Bayesian mixture models to help resolve ambiguous taxonomic ambiguities [56]. This is a strength as easily communicated methods will aid interpretation of metagenomics results by the clinical workforce.

The relatively low sensitivity for pathogen detection through metagenomic sequencing compared to other diagnostic tools such as culture and specific molecular tests has also held back implementation into clinical practice [29]. In this study we chose to use the cell-free fraction of CSF, previously shown to provide pathogen nucleic acid enrichment in blood and CSF [35, 56–58]. We observed significant enrichment for pathogen-specific information in the cell-free CSF fraction, with increased RPM and coverage breadth for most species detected, reinforcing recent findings on the utility of cfDNA-based diagnostics [57, 58] For example, *N. meningitidis* achieved significantly higher genome coverage in the cell-free fraction. Higher genome coverage facilitates strain typing, identification and antibiotic resistance gene determination. The ability to obtain genome information is a benefit of metagenomic sequencing and has implications for public health reporting and antibiotic stewardship. Levels of human DNA in samples with high cell counts have the potential to limit genome recovery even in the cell-free fraction, potentially masking pathogen reads. *Streptococcus pneumoniae* was the only bacterial assignment which had a higher genome coverage in the cell pellet fraction, this sample had a high cell count and the highest-host background in the sample set. This observation is consistent with known interference challenges in high-host backgrounds [59]. Cellularity in CSF samples is highly variable and likely determined by host response and pathogen burden. Due to the small number of samples in this study the true effect of cellularity is hard to determine, we can observe using our data set that cell count and read numbers are not simply correlated and genome recovery can be seen to be independent of both.

While RNA viruses have successfully been detected in CSF using metagenomic techniques [23, 60], we believe the inclusion of SMART-9 N enhanced detection of RNA viruses in the cell-free fraction of CSF and is a promising route for follow-up studies. Enterovirus was missed in both fractions in one patient and in each fraction for two patients suggesting that there is potential to optimise this method.

Discrepancy in reporting of viruses between metagenomics and clinical microbiology could be due to a low infectious burden, low number of sequencing reads, as seen in the cell free fraction of patient five, high host background as seen in the cell pellet fraction of patient 30 or due to degradation of the virus prior to metagenomic sequencing.

Metagenomic sequencing provides an opportunity to transform clinical microbiology with actionable, high-resolution pathogen data. These data suggest a promising diagnostic sensitivity profile matching or exceeding the pathogen information available from traditional microbiology methods for many pathogens. However, as with traditional microbiology there is a need for a dedicated MDT approach incorporating clinical scientists, microbiologists, neurologists and bioinformaticians to contextualise results. Discordant results in our dataset such as identifying *Staphylococcus spp.* in a sample which had been reported as containing Enterovirus by the clinical microbiology laboratory would require careful review before being decided clinically relevant. *S. warnerii* was reported by the clinical microbiology laboratory by enrichment culture, this was not detected using metagenomics, the organism could be below the level of detection for metagenomics or could represent a contaminant introduced in the microbiology laboratory. A clinical review would be required to establish the significance of any discordant finding. Similarly, the detection of Human herpes viruses in the cell free fraction of several samples is of unknown clinical significance, it is not possible to determine using metagenomics alone if these are linked to latent or active infection.

Our finding that two OND samples showed hits to organisms in the allowed taxon whitelist in the cell-free fraction (*C. acnes* and *S. epidermidis*) further highlights the promises and peril of metagenomic diagnostic approaches and the need for consensus-driven interpretation as these are also common contaminants [19, 26, 61].

A limitation of this study is the low number of samples obtained from different patients. In addition, the ‘gold standard’ of conventional microbiology has a low rate of microbiological diagnosis, only 53% in this study. For these reasons it is inappropriate to calculate sensitivity or specificity of the assay compared to conventional microbiology techniques or syndromic diagnosis of CNSI. Our proof of concept study indicates that the cell free fraction of CSF has the potential to add value to CSF metagenomics and warrants further studies to fully establish clinical use. Our findings are supported by prior studies showing that cell-free DNA (cfDNA) approaches achieve higher pathogen sensitivity compared to conventional microbiology, even under conditions of high host DNA background [56–58, 61].

## Conclusion

In summary, we present a metagenomic sequencing workflow encompassing simple pathogen enrichment and a practical yet effective bioinformatic analysis step. Methods such as this have potential for integrating metagenomic sequencing into routine CNSI diagnostics, suitable for laboratories looking to adopt this technology in a practical, clinically relevant way. While composite filtering measures and cell-free enrichment have improved our detection specificity, real-world implementation will require regular interdepartmental review and larger studies to fully optimise sensitivity and specificity thresholds. Development of a reproducible framework also provides a foundation for future consensus and standardisation efforts in clinical metagenomic sequencing, with the aim to bring this vision closer to everyday clinical practice.

## Supplementary Information

Below is the link to the electronic supplementary material.

Supplementary Material 1

Supplementary Material 2

Supplementary Material 3

Supplementary Material 4

Supplementary Material 5

## Data Availability

The datasets generated during the current study are available in the European Nucleotide Archive (ENA) at EMBL-EBI repository under accession number PRJEB81291 following host de-contamination ([https://www.ebi.ac.uk/ena/browser/view/PRJEB81291](https:/www.ebi.ac.uk/ena/browser/view/PRJEB81291)).

## Abbreviations

BIA: British Infection Association
cfDNA: Cell-free DNA
CNSI: Central Nervous System Infections
CSF: Cerebrospinal Fluid
DNA: Deoxyribonucleic Acid
ECMV: Encephalomyocarditis Virus
HBRC: Human Biomaterials Resource Centre
HHV-7: Human Herpesvirus 7
HHV-4: Human Herpesvirus 4
ND: Nothing Detected
OND: Other Neurological Disease (Non-infectious control cases)
ONID: Other Neuroinflammatory Disease
RNA: Ribonucleic Acid
RPM: Reads Per Million
SURPI+: Sequence-based Ultra-Rapid Pathogen Identification+
WHO: World Health Organization
